# Diffuse large B-cell lymphoma: examining evolving patterns in mortality, incidence, and demographics

**DOI:** 10.1007/s12094-025-03859-4

**Published:** 2025-02-18

**Authors:** Silpa Choday, Eric Tran, Miguel Gonzalez

**Affiliations:** 1 Department of Internal Medicine, Creighton University School of Health Science, Phoenix, AZ USA; 2 Department of Hematology/Oncology, Dignity Health Medical Group, Phoenix, AZ USA

**Keywords:** Diffuse large B-cell lymphoma, DLBCL, Non-Hodgkin lymphoma, Trends, Hospitalization

## Abstract

**Background:**

Diffuse large B-cell lymphoma (DLBCL) is the most common subtype of non-Hodgkin lymphoma, characterized by its aggressive nature and heterogeneity. This study analyzes recent trends in DLBCL including trends in hospitalization, inpatient mortality, and costs.

**Methods:**

Using the Nationwide Inpatient Sample (NIS) database from 2016 until 2020, a retrospective cohort study was performed to identify DLBCL hospitalization, discharges, and investigate outcomes. Trends were adjusted for age, sex, race, insurance type, mean household income, and hospital characteristics. Multivariable logistic regression has been used to analyze the data.

**Results:**

A total of 103,588,729 records were analyzed, identifying 47,425 cases with a diagnosis of DLBCL. From 2016 to 2020, hospitalizations have increased from 14,980 to 16,565. The mean age at diagnosis was 65 (*P* < 0.001). Males were slightly more affected than females (57.3 vs 42.6), with an increasing trend in males from 53.7% to 62.3% (*P* = 0.03). The highest prevalence was observed in the White population, followed by Hispanics and African Americans. Notably, the prevalence among Hispanics increased from 10 to 12%, while there is a decreasing trend in other demographics (*P* = 0.05). Medicare was the most common insurance, with increasing trends, followed by Medicaid and private insurance (*P* = 0.6). Inpatient mortality increased from 6.1 to 7.1 (2016 to 2018) and decreased to 6.1% (2018 to 2020) (*P* < 0.001). The mean length of the stay remained stable at 11.8 days. However, hospital charges increased from $176,131 to $212,324. Comorbidities such as obesity, hypertension, other associated lymphomas, peripheral vascular diseases, and diabetes showed an increasing trend (*P* < 0.05). Discharges to home and skilled nursing facility (SNF) decreased, while there was an increase in discharges to home with home health (HH) care and short-term care (*P* < 0.001).

**Conclusion:**

Risk factors for DLBCL include white male sex, with the mean age of 65 years. The incidence among the Hispanic population has been increasing over the years. There are disparities in incidence and survival among different ethnic/demographic groups that need to be addressed by identifying targeted interventions and equitable healthcare access.

## Introduction

Diffuse large B-cell lymphoma (DLBCL) is a highly heterogeneous and aggressive hematologic malignancy that represents the most common subtype of non-Hodgkin lymphoma (NHL) [1]. The standard of care for newly diagnosed DLBCL is R-CHOP: consisting of rituximab, cyclophosphamide, doxorubicin, vincristine sulfate, and prednisone [14]. Approximately 30% to 40% of patients with DLBCL experience a relapse, while 10% are resistant to first-line treatments [2]. DLBCL accounts for about a third of all NHL cases globally, with prevalence varying between 20 and 50% depending on the country [3]. In the United States, the age-standardized incidence rate for DLBCL is 7.2 per 100,000, with rates increasing with age and being generally higher in males than females [3]. Among racial groups, the incidence is highest in non-Hispanic whites, at 9.2 per 100,000 [3]. This article examines trends in DLBCL, including mortality rates, incidence, and demographic differences.

## Methods

This study analyzed hospitalizations principally for DLBCL in the US. The complete dataset was obtained from the NIS spanning 2016 to 2020. This extensive, comprehensive database encompasses national hospital discharges. It is meticulously maintained as an integral component of the healthcare cost and utilization project (HCUP) by the agency for healthcare research and quality (AHRQ). This sample includes community, general hospitals, and academic medical centers but excludes long-term facilities. Hospitals were divided based on geographic region, urban vs. rural location, teaching status, ownership, and number of beds. Each record in the NIS represents a single hospital discharge and includes a unique identifier, demographic data (age, gender, and race), hospital transfer status, admission type (emergent, urgent, or elective), primary and secondary diagnoses (up to 15), primary and secondary procedures (up to 15), expected primary and secondary insurance payers, total hospital charges, LOS, and hospital characteristics (region, urban vs. rural location, bed-size and teaching status).

## Outcomes of the study

A retrospective analysis was conducted on a dataset containing 103,588,729 records, identifying 47,425 cases with a diagnosis of DLBLC between 2016 and 2020. The study examined temporal trends in hospitalizations, demographic characteristics, comorbidities, mortality, and healthcare utilization. Key variables included patient age, sex, race, comorbidities, hospitalization metrics, and discharge outcomes. Changes over time were assessed using statistical tests to identify significant trends.

*Demographics* Trends in age, sex distribution, and racial demographics were analyzed. The mean age of diagnosis and the proportion of males versus females were calculated, with a focus on changes over the study period.

*Comorbidities* Prevalence rates for diabetes complications, hypertension, obesity, peripheral vascular disease (PVD), drug use, smoking, heart failure, renal failure, alcohol use, cancer, and coagulopathy were examined.

*Hospitalization metrics* Inpatient mortality rates, mean length of stay, and inflation-adjusted hospital charges were analyzed to identify significant changes.

*Discharge outcomes* Trends in discharge destinations, including discharge to home with home health (HH), short-term care, home, and skilled nursing facilities (SNFs), were evaluated.

**Fig. 1:**
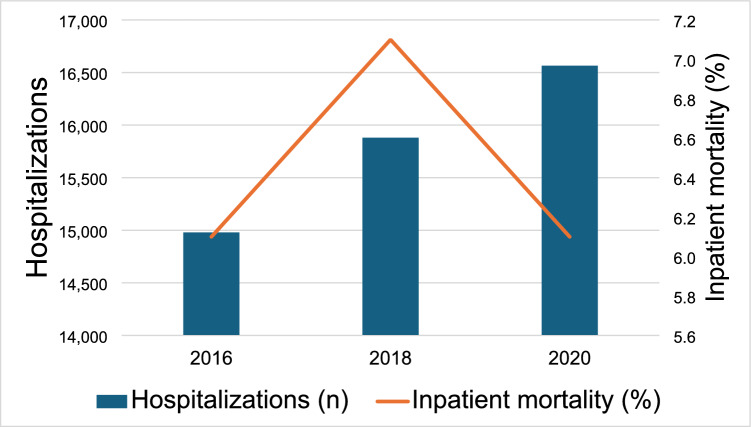
Yearly trends of hospitalizations and inpatient mortality

**Fig. 2:**
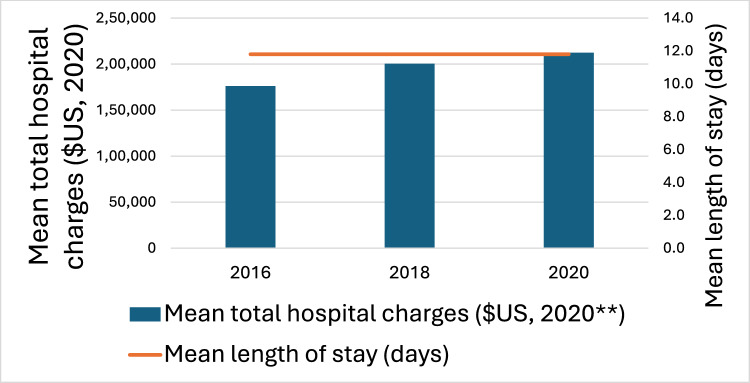
Health care utilization trends

Statistical significance was determined using appropriate tests (e.g., Chi-square, *t* tests), with a threshold of P < 0.05. Results were stratified by year to highlight temporal trends and presented in tables and figures for clarity (Table 1), (Figs. 1, 2).

**Table 1:**
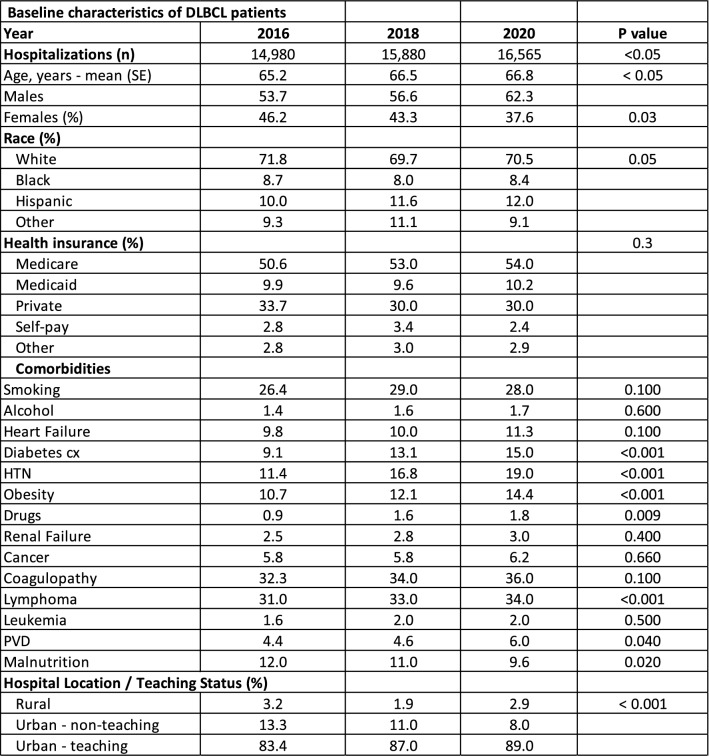
Baseline characteristics

A multivariate regression analysis with STATA was used to calculate the trend in mortality, length of stay (LOS), and total hospital costs (THC) following adjustment for age, sex, race, grouped with Charlson comorbidity index, insurance type, mean household income, and hospital characteristics. The calculation of total hospital costs involved utilizing HCUP cost-to-charge ratio files. Institutional review board approval was not needed for this database study.

## Results

A total of 103,588,729 records were analyzed, identifying 47,425 cases with a diagnosis of DLBCL. From 2016 to 2020, hospitalizations have increased from 14,980 to 16,565. The mean age at diagnosis was 65 (*P* < 0.001). Males were slightly more affected than females (57.3 vs 42.6), with an increasing trend in males from 53.7% to 62.3% (*P* = 0.03) [Table 1]. There were significant differences in racial demographics over the years (*P* < 0.001), with consistent proportions of White (70%), Black (8%), Hispanic (11.5%), and other races (10%). Notably, the prevalence among Hispanics has increased (*P* = 0.05). There is an increased usage of Medicare insurance (50.6% to 54.0%) and Medicaid (9.9% to 10.2%) with decreased usage of private (33.7% to 30.0%) and self-pay (2.8% to 2.4%) (*P* = 0.3).

There are several comorbidities that showed significant changes. There is an increased prevalence of diabetes complications (9.1% to 15.0%, *P* < 0.001), HTN (11.4% to 19.0%, *P* < 0.001), obesity (10.7% to 14.4%, *P* < 0.001), drugs (0.9% to 1.8%, *P* = 0.009), PVD (4.4% to 6.0%, *P* < 0.001). There was no significant change in the prevalence of smoking, heart failure, alcohol, renal failure, cancer, and coagulopathy (*P* > 0.05) [Table 1].

Inpatient mortality rates stayed at 6.1% (*P* < 0.001) (Fig. 1). The mean length of hospital stay was at 11.8 days (*P* < 0.001) (Table 2). Mean total hospital charges increased significantly from $176,131 in 2016 to $212,324 in 2020 (*P* < 0.001), adjusted for inflation to 2020 USD) (Fig. 2). The percentage of patients discharged to home with HH significantly increased from 19.2% to 24.7% (P < 0.001), slight increase in DC to short-term care from 2.7% to 3.1% (*P* < 0.001). Discharged to home and SNF has decreased (*P* < 0.001).

**Table 2:**
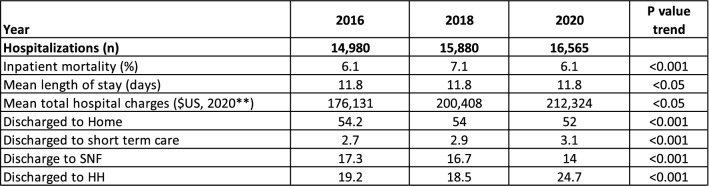
Outcomes of hospitalizations

## Discussion

This discussion focuses on the analysis of hospitalization data spanning from 2016 to 2020, with epidemiological trends, patient demographics, healthcare utilizations, clinical outcomes, and healthcare disparities. DLBC cancer remains one of the most common lymphomas and accounts for one third of non-Hodgkin lymphoma cases in the developed world [4]. Across all continents, DLBCL is the most common NHL subtype, ranging from 30 to 54%. In the United States, the incidence rate is 7.2 per 100,000, primarily affecting non-Hspanic whites. Several established risk factors contribute to the development of DLBCL, including prior cancer history, B-cell activating autoimmune diseases, HCV, and obesity [5].

This study analyzed a large dataset comprising over 103 million records from 2016 to 2020, identifying 47,425 cases of DLBCL. The mean age of diagnosis increased from 65.2 to 66.8 from 2016 to 2020. Hospitalization increased dramatically, from 14,980 in 2016 to 16,565 in 2020, increasing by over 10%. We discovered that the most common comorbidities among DLBCL patients included coagulopathy, lymphoma, smoking, hypertension, and obesity. Likewise, a 2017 study utilizing the Charlson Comorbidity Index (CCI) found that patients with a CCI score of three or higher had a significantly lower 5-year overall survival rate compared to those with a CCI score of less than two. In addition, patients with higher CCI were predicted to require dose reductions due to adverse events, higher hospitalization rates, and higher relapse rates [6]. A similar 2023 study analyzed 80,930 DLBCL hospitalizations and found an inpatient mortality of 6.56%. The mean age at death was 67.99 years, compared to 64.90 years for those who survived. In addition, a CCI score of 3, 4, and ≥ 5 was associated with increased odds of mortality by 35%, 43%, and 53%, respectively [8, 16]. Moreover, patients who developed complications such as tumor lysis syndrome, pancytopenia, congestive heart failure (CHF), atrial fibrillation, and acute kidney injury during hospitalization had significantly higher odds of inpatient mortality, with increases of 87%, 104%, 36%, 75%, and 295%, respectively [16].

A study in 2018 analyzed the hospitalization and health care costs of patients with DLBCL and FL found that majority of the medical care costs consisted of chemoimmunotherapy and supportive care costs, with 80% and 71% of patients requiring chemoimmunotherapy and supportive care pharmacy prescription fills, respectively [11]. Medicare continues to be predominant insurance provided with a decrease in usage of private insurance. Of patients diagnosed with DLBCL with Medicare, pharmaceutical agents continue to be the bulk of the healthcare costs. As patients progressed to second-, third-, and even fourth-line treatment, inpatient costs increased, suggesting a more significant lymphoma burden and increased inpatient requirements [12]. Furthermore, a disparity in treatment of frail and elderly patients with Medicare was noted, up to 33% of patients not receiving appropriate treatment. The increased usage of Medicare and increasing age of DLBCL diagnosis suggest a lack of adequate treatment in the elderly, highlighting a major disparity in elderly patients [13].

In our study, we saw hospitalization costs increase by more than 20% from $176,131 in 2016 to $212,324 in 2020 while the mean length of hospitalization staying consistent at 11.8 days. This is in part due to the expensive costs of first-line DLBCL treatment, and the exorbitant costs of treatment for relapsed DLBCL. The increased costs can be due to starting chemotherapy inpatient for the first cycle or if the patient had any complications and must be admitted for inpatient chemotherapy. The mainstay of treatment for newly diagnosed DLBCL is R-CHOP: consisting of rituximab, cyclophosphamide, doxorubicin, vincristine sulfate, and prednisone. R-CHOP is typically used as first-line therapy for all patients from stage I to IV of DLBCL [15]. A study in 2023 analyzed the hospitalization and treatment costs of DLBCL in patients with first-line treatment and second line and subsequent treatment between October 2015 and December 2018 in 66 years or older. Their data showed that first-line treatment of R-CHOP or similar regimen averaged $84,416 over a 12-month period. In 2016, patients in the evaluation phase and diagnosed with DLBCL and treated with R-CHOP therapy, the health care costs equated to $127,202. In April 2023, Pola-R-CHP (Polatuzumab vedotin, rituximab, cyclophosphamide, doxorubicin, prednisone) was approved by the FDA for previously untreated DLBCL. Pola-R-CHP had a 76.7% chance of survival without progression at 28.2 months compared to 70.2% of R-CHOP. However, survival at 2 years did not differ significantly between the groups [19]. Pola-R-CHP had a cost of $70,719/QALY compared to $88,855/QALY of R-CHOP [20].

If DLBCL is refractory to first-line treatment or DBLCL relapses, then the patient is eligible to receive second-line treatment, consisting of CAR T-cell therapy or stem cell transplant [7]. Among patients who relapsed or were refractory to R-CHOP therapy, the health care costs were even greater, totaling $164,631 for incident relapse patients [9]. Hematopoietic cell transplant requires G-CSF (granulocyte-colony stimulating factor) and plerixafor therapy, totaling a mean of $104,000 USD in 2019. Allogenic hematopoietic cell transplant totaled a mean of $490,787 in 2019 per year per patient [18].

In 2018, two CAR T-cell therapies were approved for relapsed/refractory DLBCL: axicabtagene ciloleucel and tisagenlecleucel, both which total $373,000. These two therapies utilize T cells to bind CD19 to B cells to trigger an immune response to the lymphoma. In a landmark study, Bishop et.al compared tisagenlecleucel treatment response with standard care in patients with high-grade diffuse large B-cell lymphoma. They determined that tisagenlecleucel was not superior to the standard treatment of salvage chemotherapy, which typically consists of platinum-based immunochemotherapy, with high-dose chemotherapy and subsequent hematopoietic stem cell transplantation [5]. For reference, salvage chemotherapy would be $105,000/QALY compared to $284,000 QALY for CAR T-cell therapy [10]. Another study, the international phase 3 trial ZUMA-7 demonstrated superior outcomes in patients treated with axicabtagene ciloleucel compared to those receiving standard therapy, which included chemoimmunotherapy and high-dose chemotherapy with autologous stem cell transplantation [17]. Patients in the axicabtagene ciloleucel group showed an 83% overall response rate, with 65% achieving a complete response, while the standard therapy group had a 50% overall response rate and a 32% complete response rate [17]. However, the incidence of adverse events of grade 3 + was higher in the axicabtagene ciloleucel group, occurring in 91% of patients, compared to 83% in the standard therapy group (17).

## Conclusion

In conclusion, this study provides a comprehensive analysis of DLBCL hospitalizations from 2016 to 2020, highlighting significant trends in patient demographics, comorbidities, and healthcare costs. The increasing hospitalization rates, particularly among males and Hispanics, alongside the rising costs of treatment reflect the growing burden of DLBCL on the healthcare system. Despite stable hospitalization durations, the economic impact of treating DLBCL, especially in cases requiring advanced therapies like CAR T-cell treatments, is substantial. These findings emphasize the need for continued research and strategies to optimize care and manage the rising costs associated with this prevalent lymphoma.

## Data Availability

Data have been collected from PubMed and google scholar.

## Abbreviations

CCI: Charlson comorbidity index
CAR T: Chimeric antigen receptor T-cell
DLBCL: Diffuse large B-cell lymphoma
HCV: Hepatitis C virus
LOS: Length of stay
THC: Total hospital costs
SNF: Skilled nursing facility
HH: Home with home health
NHL: Non-Hodgkin lymphoma
NIS: National Inpatient Sample
R-CHOP: Rituximab, cyclophosphamide, doxorubicin (hydroxydaunomycin), vincristine (oncovin), prednisone
